# PSSA-2, a Membrane-Spanning Phosphoprotein of *Trypanosoma brucei*, Is Required for Efficient Maturation of Infection

**DOI:** 10.1371/journal.pone.0007074

**Published:** 2009-09-17

**Authors:** Cristina M. Fragoso, Gabriela Schumann Burkard, Michael Oberle, Christina Kunz Renggli, Karen Hilzinger, Isabel Roditi

**Affiliations:** 1 Institut für Zellbiologie, Universität Bern, Bern, Switzerland; 2 Swiss Tropical Institute, Basel, Switzerland; Louisiana State University, United States of America

## Abstract

The coat of *Trypanosoma brucei* consists mainly of glycosylphosphatidylinositol-anchored proteins that are present in several million copies and are characteristic of defined stages of the life cycle. While these major components of the coats of bloodstream forms and procyclic (insect midgut) forms are well characterised, very little is known about less abundant stage-regulated surface proteins and their roles in infection and transmission. By creating epitope-tagged versions of procyclic-specific surface antigen 2 (PSSA-2) we demonstrated that it is a membrane-spanning protein that is expressed by several different life cycle stages in tsetse flies, but not by parasites in the mammalian bloodstream. In common with other membrane-spanning proteins in *T. brucei*, PSSA-2 requires its cytoplasmic domain in order to exit the endoplasmic reticulum. Correct localisation of PSSA-2 requires phosphorylation of a cytoplasmic threonine residue (T_305_), a modification that depends on the presence of TbMAPK4. Mutation of T_305_ to alanine (T_305_A) has no effect on the localisation of the protein in cells that express wild type PSSA-2. In contrast, this protein is largely intracellular when expressed in a null mutant background. A variant with a T_305_D mutation gives strong surface expression in both the wild type and null mutant, but slows growth of the cells, suggesting that it may function as a dominant negative mutant. The PSSA-2 null mutant exhibits no perceptible phenotype in culture and is fully competent at establishing midgut infections in tsetse, but is defective in colonising the salivary glands and the production of infectious metacyclic forms. Given the protein's structure and the effects of mutation of T_305_ on proliferation and localisation, we postulate that PSSA-2 might sense and transmit signals that contribute to the parasite's decision to divide, differentiate or migrate.

## Introduction

Throughout its digenetic life cycle in mammals and tsetse, *Trypanosoma brucei* is covered by stage-specific coats of glycosylphosphatidylinositol (GPI)-anchored molecules. The most abundant components are present in several million copies per cell and are expressed during defined windows of the life cycle. Bloodstream forms in the mammalian host are covered by a uniform coat consisting of one type of variant surface glycoprotein (VSG) at a time. This protects the parasite from destruction by the host innate immune system and allows it to evade the adaptive immune response by periodically switching to a new VSG, a process known as antigenic variation. In the midgut of the tsetse vector, as the parasite differentiates to the procyclic form, it replaces the VSG coat by GPI-anchored proteins known collectively as procyclins. These proteins are characterised by internal dipeptide (EP) or peptapeptide (GPEET) repeats. GPEET procyclin is the major component of the coat during the first few days of infection (early procyclic forms), but is replaced by EP procyclins as the trypanosome differentiates to the late procyclic form [1]. Epimastigote forms in the salivary glands have a stage-specific coat consisting of *brucei* alanine-rich proteins (BARP) [2], while metacyclic forms, which are infectious for a new mammalian host, again have a VSG coat, but draw on a different and more limited repertoire than bloodstream forms [3].

In recent years it has become apparent that the surface coats of insect forms of *T. brucei* and *T. congolense* are more similar than was previously supposed. Midgut forms of *T. congolense* express procyclins with characteristic heptapeptide (EPGENGT) repeats [4], while epimastigotes express glutamic acid/alanine-rich proteins [5], [6] that are related to *T. brucei* BARPs. Two additional surface molecules have been identified in *T. congolense*, a protease-resistant surface molecule [7] that is expressed by early procyclic forms and a newly discovered GPI-anchored protein, *congolense* epimastigote-specific protein (CESP) [8]. Genes encoding proteins related to CESP are also found in *T. brucei (Tb*927.8.930, 950 and 970). Epimastigote forms of the two species exhibit different tissue tropisms in the fly, with *T. brucei* colonising the salivary glands and *T. congolense* colonising the proboscis. At present it is not known which parasite molecules determine this.

The abundance of the major surface molecules has impeded the identification of other membrane proteins, so that relatively little is known about minor components of the parasite coat. Two families of invariant surface proteins of unknown function, ISG65 and ISG75, are expressed by bloodstream forms, but not by procyclic forms [9]. Glycoconjugates on the surface of procyclic forms have been described by Güther at al [10]. These are also found in cells deficient in *TbGPI12*, implying that they do not possess GPI anchors. Of the few membrane-associated proteins characterised in procyclic forms, three are enzymes that modify the surface. Zinc metalloproteases of the MSP-B family, which are expressed during the differentiation of bloodstream to procyclic forms and in established procyclic forms, are involved in VSG shedding [11]. In procyclic forms, a trans-sialidase transfers sialic acid from extracellular donor molecules to the GPI anchors [12], [13]. Both of these proteins are predicted to be GPI-anchored, but they are likely to be of low abundance since they could not be detected by biosynthetic labelling, even in cell lines devoid of procyclins [14], [15]. A membrane-associated kinase that phosphorylates threonines in the pentapeptide repeat of GPEET is active in early procyclic forms, but not in bloodstream forms, late procyclic forms or epimastigotes [15], [16]; neither the protein nor the gene has been identified. Downstream of the procyclin genes, and forming part of the same polycistronic transcription units, are several procyclin-associated genes that are also stage-regulated, with increased levels of mRNA in procyclic forms [17], [18]. All of these potentially encode secreted or membrane proteins, but as yet there is no evidence that the proteins are produced, and none of the genes is essential for transmission through tsetse [19].

Sixteen years ago, COS cell expression cloning combined with antibody panning was used to identify surface molecules of procyclic forms [20]. Of the two cDNA clones that were isolated, one encoded EP1 procyclin, confirming the validity of the approach. The other clone, PSSA-2, was predicted to encode a membrane-spanning protein with a C-terminal domain consisting of degenerate proline- and tyrosine-rich repeats. PSSA-2 mRNA was more abundant in procyclic forms, suggesting that it might be stage-regulated, but there was no information on the expression of the protein. Here we demonstrate that the localisation and topology of PSSA-2 are as postulated, and that the cytoplasmic tail is required for correct targeting of the protein. PSSA-2 null mutants have no perceptible phenotype in culture, but are compromised in their ability to produce mature infections in tsetse.

## Results

### PSSA-2 is localised on the surface of procyclic forms

**Figure 1:**
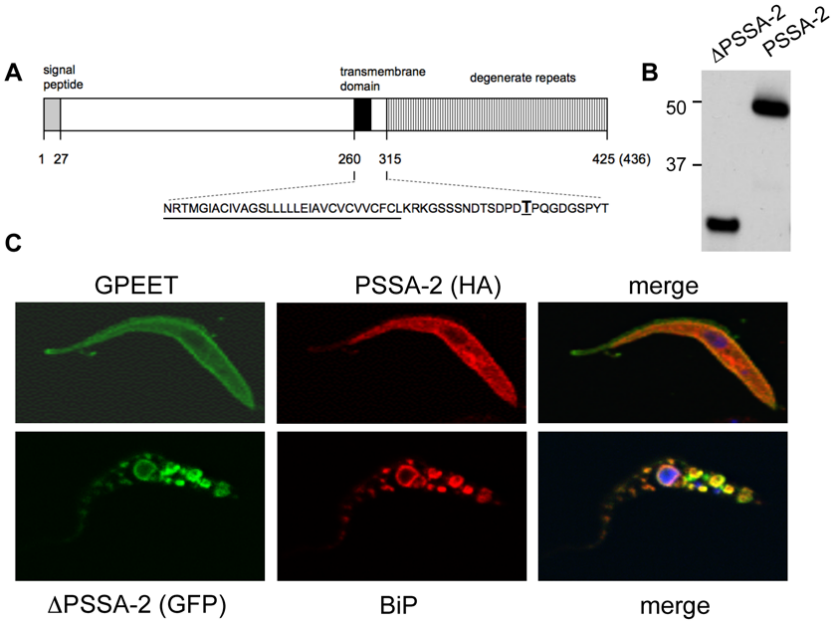
PSSA-2 requires the cytoplasmic tail for surface localisation. A. Schematic representation of PSSA-2. Predicted structural domains and signal peptide are indicated (not drawn to scale). AnTat 1.1 encodes a polypeptide of 436 amino acids, whereas the genome strain TREU 927/4 encodes a polypeptide of 425 amino acids. The sequence corresponding to the transmembrane domain is underlined; threonine residue T_305_ is in boldface type and underlined. B. Immunoblot analysis of total lysates from procyclic forms of AnTat 1.1 stably transfected with plasmids encoding either an HA-tagged version of truncated PSSA-2 (ΔPSSA-2), lacking the cytoplasmic domain from residues 292–436, or full-length PSSA-2. Proteins were detected with an anti-HA antibody. 10^6^ cell equivalents were loaded per lane. Markers are indicated on the left. C. Immunofluorescence analysis of HA-tagged full length PSSA-2 (top panel) and a ΔPSSA-2/GFP fusion protein (lower panel). Trypanosomes were fixed with formaldehyde and glutaraldehyde, permeabilized with Triton X-100 and stained with anti-GPEET, anti-HA or anti-BiP antibodies as indicated.

PSSA-2 is encoded by a single copy gene in *T. brucei* (Tb10.26.0790) and shares 64% identity to a protein in *T. congolense* and 49% to a protein in *T. vivax*. Amplification of the coding region of PSSA-2 from *T. brucei* AnTat 1.1 revealed small differences to the sequence in GeneDB, the most prominent of which was an insertion of 30 base pairs encoding an additional tyrosine/proline-rich repeat. PSSA-2 was previously predicted to consist of an N-terminal extracellular domain, a single membrane-spanning domain and a C-terminal cytoplasmic domain containing several copies of a YGQP motif [20]. To analyse this, we first attempted to produce antisera against different domains of PSSA-2, expressed as bacterial fusion proteins. Although these antisera recognised the recombinant protein moieties, they did not bind procyclic forms in IFA or recognise a protein on immunoblots (data not shown). We therefore used green fluorescent protein (GFP) or a haemagglutinin (HA) tag to localise two versions of the protein, a full-length form and a truncated form (^Δ292–436^PSSA-2) that lacked the predicted cytoplasmic domain (Fig. 1). Western blot analysis of stable transformants expressing the HA-tagged full length and truncated proteins detected bands of 50 kDa and 35 kDa, respectively, consistent with the predicted sizes of the polypeptides (Fig. 1B). In cells in which the full-length protein was tagged at the C terminus, PSSA-2 was detected on the surface, colocalising with GPEET (Fig. 1C). Detection of the HA tag required permeabilisation of the cells with Triton X-100, indicating that the C-terminal of the protein was indeed cytoplasmic as predicted. In contrast, the truncated version was retained in the endoplasmic reticulum, colocalising with BiP (Fig. 1C). Replacing GFP by an HA tag did not alter the localisation (data not shown). These results indicate that the cytoplasmic tail is required for correct targeting to the plasma membrane.

### Expression of PSSA-2 during the trypanosome life cycle

**Figure 2:**
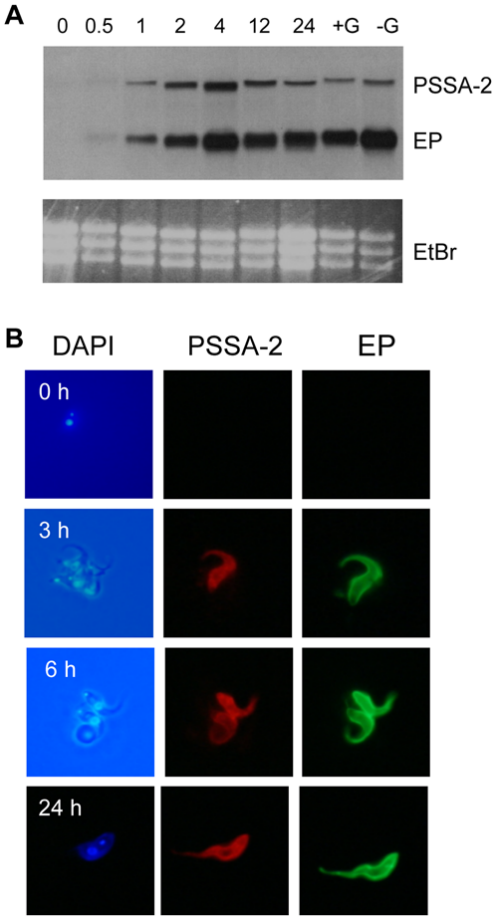
Detection of PSSA-2 and procyclin during synchronous differentiation of stumpy bloodstream forms to procyclic forms. A. Northern blot analysis of wild type AnTat 1.1. Numbers denote hours after triggering differentiation. +G: early procyclic forms cultured in the presence of glycerol. −G: late procyclic forms cultured without glycerol. EtBr: ethidium bromide-stained rRNA is shown as a loading control. B. Immunofluorescence analysis of cells expressing HA-tagged PSSA-2 during differentiation from bloodstream forms to procyclic forms. At given time points after differentiation was triggered, cells were fixed and stained with DAPI (left panels), anti-HA to detect PSSA-2 (central panels) and anti-EP (right panels).

The original publication on PSSA-2 demonstrated that the messenger RNA was more abundant in procyclic forms than in bloodstream forms [20]. To analyse the developmental regulation in more detail, RNA was isolated during the first 24 h of synchronised differentiation from stumpy bloodstream forms to procyclic forms. Northern blot analysis with probes for PSSA-2 and EP procyclin (Fig. 2A) revealed that the transcripts accumulated with the same kinetics, peaking ∼4 h after differentiation was triggered and then declining in established procyclic forms. No differences in PSSA-2 mRNA levels were observed when early and late procyclic forms were compared (Fig. 2A, lanes +G and −G, respectively). To monitor the kinetics of protein expression, we used the PSSA-2-HA cell line described above, in which the tagged copy of the coding region is followed by the PSSA-2 3′ untranslated region (UTR) and intergenic region. Immunofluorescence analysis using anti-EP procyclin and anti-HA antibodies showed that neither protein was expressed by bloodstream forms (Fig. 2B). Three hours after being triggered to differentiate, two populations were observed: cells that were positive for both EP and PSSA-2, and cells that were negative for both proteins. From 6 h onwards, all cells were double-positive.

Untranslated regions can be fused to reporter genes in order to investigate expression in life cycle stages that cannot be cultured (e.g. epimastigote forms). In the constructs routinely used in our laboratory, transcription is driven by the procyclin promoter; this results in robust expression that does not interfere with regulation by the 3′ UTR, as was previously demonstrated for BARP [2]. In initial experiments, trypanosomes were stably transformed with the coding region for dsRED, followed by the 3′ UTR and intergenic region of PSSA-2. One clone was used to infect tsetse and monitored at various time points up to the production of metacyclic forms. In these experiments, all life cycle stages in the insect, with the exception of metacyclic forms, showed strong fluorescence (data not shown). This gave us the first indication that the protein might be widely expressed throughout the life cycle. The negative result obtained with metacyclic forms must be treated with caution, as there are indirect indications that genes under the control of procyclin promoters may not be active in this stage (C. Fragoso, unpublished data)[21].

**Figure 3:**
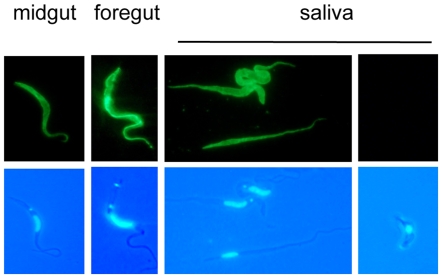
Expression of PSSA-2 in tsetse-derived trypanosomes. Immunofluorescence analysis of PSSA-2-HA expression by trypanosomes isolated from the midgut, foregut and saliva probes 28–35 days after the infective bloodmeal. Trypanosomes were fixed with formaldehyde and glutaraldehyde and stained with anti-HA antibody (upper panel) and DAPI (lower panel).

In addition to the UTRs, it is known that coding regions can provide an additional layer of regulation. This was previously observed for the procyclin transcripts that are present in salivary gland trypanosomes, but apparently not translated [21]. To take this into account we used the PSSA-2-HA cell line described above to infect tsetse. When flies were dissected 28–35 days post infection, all cells in the midgut and foregut were positive for PSSA-2, as were epimastigotes in saliva probes (Fig. 3). Once again, metacyclic forms were negative.

### A PSSA-2 null mutant shows reduced maturation of infections

Since PSSA-2 appears to be expressed during a large part of the trypanosome life cycle in the fly, and is at the interface between the trypanosome and its environment, we determined whether it was possible to generate a null mutant in procyclic forms. This was readily achieved in two rounds of transformation in which the PSSA-2 coding region was replaced by two selectable markers. When monitored for growth, the null mutant grew at the same rate as the wild type (see below).

**Figure 4:**
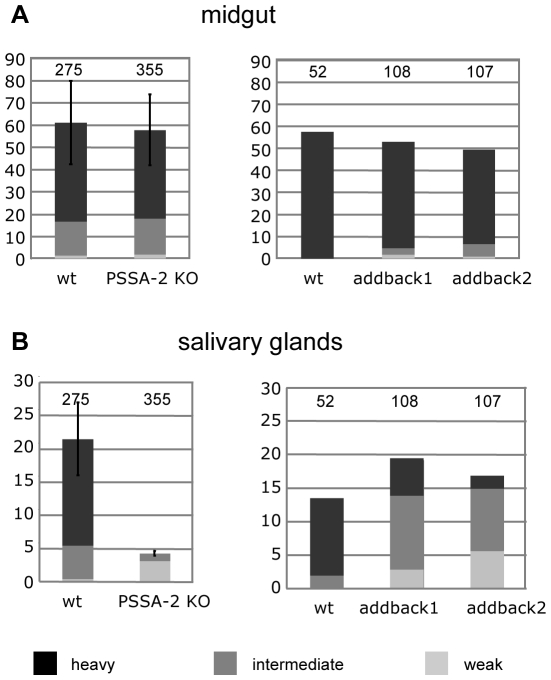
PSSA-2 does not influence midgut infections in tsetse, but is required for efficient colonisation of the salivary glands. Teneral flies were infected with procyclic forms of AnTat 1.1 (wild type), the PSSA-2 null mutant (PSSA-2 KO) or the independent addback clones 1 and 2. Flies were dissected 28–35 days post infection and graded for the prevalence and intensity of infections in the midgut and salivary glands. The y-axis represents the percentage of infected flies. The total number of flies is indicated above the bars on the graphs. For the panel on the left, this represents the total numbers from 4 independent experiments. Transmission index (see text) is the percentage of midgut infections giving rise to salivary gland infections.

To establish the role of PSSA-2 in transmission, teneral flies were infected with procyclic forms of Antat 1.1 or the PSSA-2 null mutant. Flies were dissected 28–35 days post infection and graded for the prevalence and intensity of infections. Both the wild type and the null mutant were capable of establishing midgut infections with the same efficiency (Fig. 4) and no significant differences were observed in their ability to produce heavy infections. In contrast, the null mutant gave rise to fewer, and much weaker, salivary gland infections than the wild type. The combined data from 4 independent experiments gave, on average, a 5-fold reduction in prevalence compared to the wild type. These differences are statistically significant (two-tailed paired Students T test, p<0.005). To be sure that the decreased prevalence of salivary gland infections was caused by the lack of PSSA-2, rather than a secondary mutation elsewhere in the genome, an ectopic copy was integrated upstream of the procyclin locus on one copy of chromosome 10 (Supplemental Figure S1a). This copy was in the same context as the HA-tagged copies described above (flanked by a EP1 promoter/5′ UTR and the PSSA-2 3′UTR/intergenic region), but was not modified by an epitope tag. Two independent clones were characterised by Northern blot analysis and shown to express 3.4-fold and 7.8-fold more PSSA-2 mRNA, respectively, than wild type AnTat 1.1 procyclic forms (Supplemental Figure S1b). When compared with the wild type for their ability to establish midgut and salivary gland infections, there were no significant differences in the prevalence of infections in both compartments (Fig. 4). However, most of the salivary gland infections by the addbacks were of intermediate intensity, compared to the preponderance of heavy infections by the wild type. When all infections were taken into account, the two addback clones gave transmission indices of 35.8% and 35.1%, respectively, compared to 23.3% by the wild type. This was reduced to 22.6% and 26.3% if only heavy and intermediate infections were taken into account. In summary, these data confirm that PSSA-2 is required for efficient maturation of salivary gland infections.

### Post-translational modification of PSSA-2

**Figure 5:**
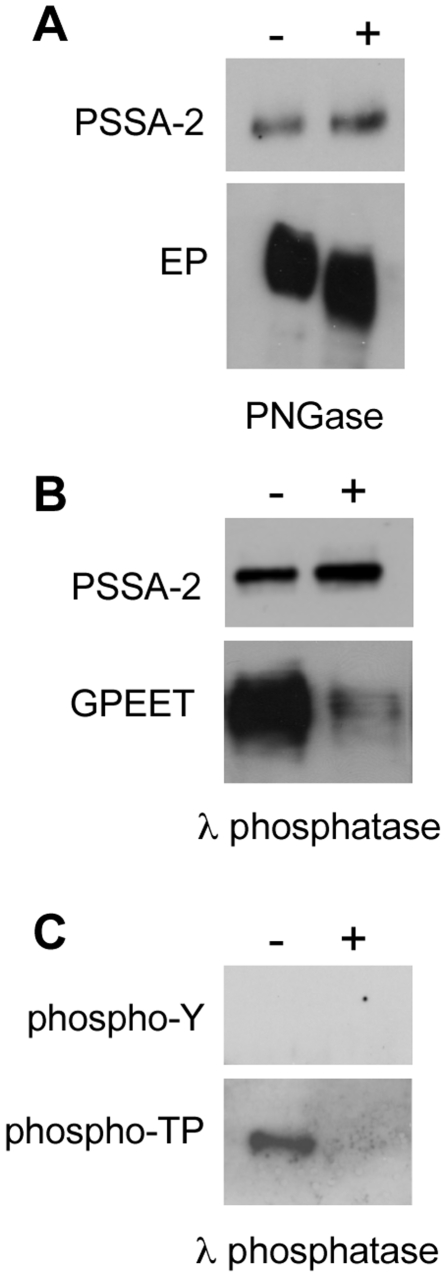
Post-translational modification of PSSA-2. A. Total lysates from procyclic forms stably expressing HA-tagged PSSA-2 were incubated in the presence (+) or absence (−) of PNGase and analysed by immunoblotting. Top panel: PSSA-2 detected with anti-HA ; lower panel, anti-EP. B. Total lysates from procyclic forms stably expressing HA-tagged PSSA-2 were incubated in the presence (+) or absence (−) of λ phosphatase and analysed by immunoblotting. MAb 5H3 (second panel) preferentially binds phosphorylated GPEET [37]. C. HA-tagged PSSA-2 was immunoprecipitated, incubated with (+) or without (−) λ phosphatase and detected with antibodies against phosphotyrosine (phosphoY; upper panel) or phosphothreonine-proline (phospho-TP; lower panel).

The PSSA-2 polypeptide contains a potential N-linked glycosylation site in the extracellular domain. To test if the protein was modified by carbohydrates, total lysates from cells expressing HA-tagged full length PSSA-2 were treated with PNGase and subsequently analysed on immunoblots. No difference in mobility was detected after treatment, suggesting that PSSA-2 is not N-glycosylated (Fig. 5A). The positive control, EP procyclin, showed a decrease in Mr confirming that the PNGase was active.

The primary sequence of PSSA-2 is rich in tyrosine, threonine and serine, and several of these amino acids are predicted to be phosphorylated. To test this, total lysates were treated with λ phosphatase, which is capable of removing phosphate groups from all three amino acids (Fig. 5B). Antibodies that discriminate between phosphorylated and non-phosphorylated forms of GPEET were used as a positive control. Although λ phosphatase treatment did not alter the mobility of PSSA-2, phosphorylation of the protein could not be excluded. In a further step, PSSA-2-HA was immunoprecipitated and probed with antibodies against phosphotyrosine (anti-pY) and phosphothreonine-proline (anti-pTP; Fig. 5C). PSSA-2-HA could be detected with anti-pTP, and reactivity was lost after treatment with λ phosphatase (Fig. 5C).

### Thr_305_ is phosphorylated and depends on a MAP kinase

**Figure 6:**
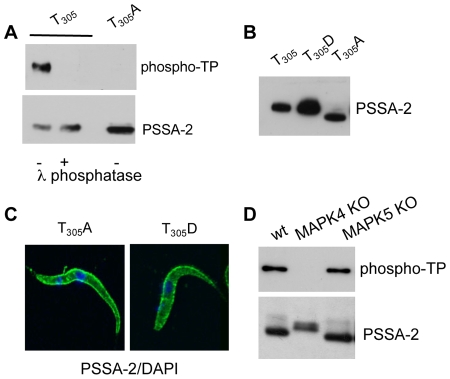
Confirmation that T_305_ is phosphorylated. A. HA-tagged PSSA-2 or PSSA-2(T_305_A) were immunoprecipated, incubated in the presence (+) or absence (−) of λ phosphatase and analysed by immunoblotting with antibodies against phospho-TP (upper panel) or anti-HA, to detect PSSA-2 (lower panel). B. Mutation of T_305_ to alanine alters the electrophoretic mobility of the protein. Total lysates from cells stably expressing PSSA-2(T_305_), PSSA-2(T_305_A) or PSSA-2(T_305_D) were detected with anti-HA antibodies. 1.5×10^5^ cell equivalents were loaded in the first two lanes and 3×10^6^ for PSSA-2(T_305_A), which is much more weakly expressed. C. PSSA-2(T_305_A) and PSSA-2(T_305_D) localise to the plasma membrane. Cells were fixed and permeablised as described in the legend to Figure 1. D. MAP kinase 4 is required for phosphorylation of T_305_. Null mutants of MAPK4 (MAPK4 KO) [23] and MAPK5 (MAPK5 KO) [22] were stably transformed with HA-tagged PSSA-2. The protein was immunoprecipated and detected with anti-phospho-TP antibodies to detect phospho-T_305_ or anti-HA to detect PSSA-2.

The primary sequence of PSSA-2 contains a single threonine (T_305_) followed by a proline residue. To confirm that this residue was phosphorylated, site-directed mutatgenesis was performed on PSSA-2-HA and the mutants were used to stably transform AnTat 1.1. Mutation of T_305_ to alanine abolished reactivity with anti-pTP (Fig. 6A) and resulted in increased migration on SDS-polyacrylamide gels (Fig. 6B). In contrast, mutation of T_305_ to aspartate, which should mimic phosphorylation, did not alter the mobility of the protein (Fig. 6B). Neither mutation had an effect on the localisation of the protein (Fig. 6C). Since treatment with λ phosphatase did not change the mobility of PSSA-2, this suggests that T_305_ might be required in order for other modifications to take place.

The amino acid sequence flanking T_305_ (DPDT_305_PQ) is a PXS/TP motif, known to be a consensus motif for MAP kinase phosphorylation. Two MAP kinase null mutants were recently generated in our laboratory [22], [23]. Procyclic forms of a MAPK4 knockout are sensitive to temperature stress [23]. Long slender bloodstream forms of the MAPK5 knockout differentiate more readily to stumpy forms, but no phenotype was detected in procyclic forms [22]. To test if phosphorylation of T_305_ is dependent on either of these kinases, MAPK4 and MAPK5 null mutants were stably transformed with PSSA-2-HA. The protein was immunoprecipitated with anti-HA matrix and probed with anti-HA and anti-pTP antibodies (Fig. 6D). When compared to expression in wild type cells, T_305_ was still phosphorylated by the MAPK5 null mutant and migrated with the same mobility. In contrast, when the protein was expressed in MAPK4-deficient cells, T_305_ was not phosphorylated. Furthermore, the anti-HA antibodies detected a doublet that migrated more slowly on polyacrylamide gels, again suggestive of altered post-translational modifications. These modifications must differ, however, from those that occur when T_305_ is mutated to alanine (compare Fig. 6B, lane T305A with Fig. 6D, lane MAPK4 KO).

### Localisation of PSSA-2 depends on T_305_ and genetic background

**Figure 7:**
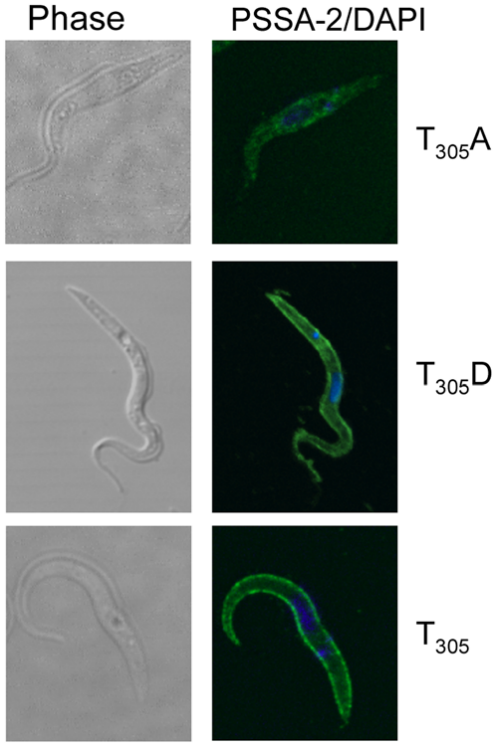
Surface localisation of PSSA-2(T_305_A) depends on the genetic background. HA-tagged wild type PSSA-2(T_305_) or the mutant forms T_305_A or T_305_D were expressed in a PSSA-2 null mutant. Left panel, phase contrast; right panel, detection of PSSA-2 with anti-HA antibodies/DAPI staining of DNA. Cells were fixed and permeablised as described in the legend to Figure 1.

**Figure 8:**
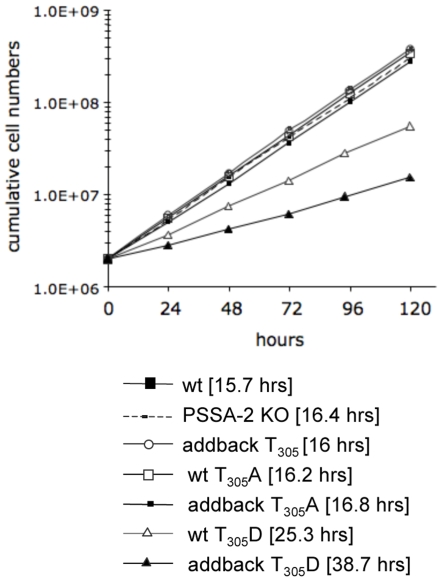
Mutation of T_305_ to aspartic acid has a deleterious effect on growth. Comparison of the population doubling time of wild type AnTat 1.1 with various PSSA-2 mutants. Cell densities were adjusted daily to 2×10^6^ cells ml^−1^ in order to ensure logarithmic growth. Generation times are shown in square brackets.

With the aim of investigating the role of T_305_ *in vivo*, we used the PSSA-2 null mutant to create a series of addbacks. In contrast to the addbacks used for the transmission experiments shown in Figure 4, these were tagged with a C-terminal HA epitope in order to detect the protein. The T_305_ addback (equivalent to PSSA-2-HA) and the T_305_D mutant both localised to the plasma membrane (Fig. 7). In contrast to what was observed in the wild type background, however, (Fig. 6C), the T_305_A variant did not show surface staining in the majority of cells (Fig. 7 and Supplemental Fig. S2) and, once again, was expressed at lower levels than the other two versions (see legend to Fig. 6B). The mislocalisation did not seem to be harmful, because this clone grew at the same rate as the wild type and the null mutant (doubling times 15.7–16.8 h; Fig. 8). The T_305_ addback also had no effect on growth. We observed, however, that the T_305_D mutation slowed growth of the cells, and that this was more pronounced in the addback, which lacked endogenous PSSA-2 (doubling time 38.7 h), than when it was expressed in a wild type background (doubling time 25.3 h). Taken together, these results suggest that T_305_ plays a role in determining the localisation of PSSA-2 and that a mutation mimicking constitutive phosphorylation might be acting as a dominant negative mutant. However, given that both mutations already affect the normal function of the protein in procyclic culture forms, we did not test them further in tsetse.

## Discussion

In this study we have demonstrated that PSSA-2 is a surface protein of insect forms of *T. brucei* with an extracellular N-terminal domain and a cytoplasmic C-terminal domain as predicted by Jackson and coworkers sixteen years ago [20]. Single transmembrane-spanning surface proteins, exemplified by ISG65 and ISG75 [24], have been identified in bloodstream forms, but this is the first example in insect forms. Such proteins could potentially interact with external factors and transduce signals to the parasite's interior, which was the original rationale for comparing full length and truncated PSSA-2. No conclusions could be drawn from this experiment, however, as removing the C-terminal domain resulted in retention of the protein in the endoplasmic reticulum. This indicates that the cytoplasmic tail contains signals for forward trafficking of the protein to the plasma membrane. In this respect PSSA-2 resembles ISG65 and CRAM, both of which require the cytoplasmic domain in order to exit from the endoplasmic reticulum [25], [26].

PSSA-2 is not expressed by bloodstream forms, but could be detected in all life cycle stages in tsetse apart from metacyclic forms. Thus, it differs from the major surface proteins, which show a more restricted expression pattern. Deletion of PSSA-2 had no effect on midgut infections, but resulted in a decrease in both the prevalence and intensity of salivary gland infections, a phenotype similar to the one obtained with a procyclin null mutant [15]. Re-expression of PSSA-2 restored the ability of parasites to colonise the salivary glands and produce metacyclic forms, although there was a shift towards infections of intermediate, rather than heavy intensity. When weak salivary gland infections were also taken into account, the two PSSA-2 addback mutants completed the life cycle slightly more efficiently than the wild type. Data on the number of metacyclic forms required to initiate an infection in mammals are scant, although experiments performed on humans bitten by tsetse suggested that several hundred parasites were needed to infect an “average man” [27]. This figure may well be an overestimate, however, since the trypanosomes were passaged in animals and some of them could have lost the property of human serum-resistance. We have observed that flies with weak salivary gland infections can readily transmit parasites to mice ([15] and the PSSA-2 null mutants in this study), and it has been documented that a single fly bite is sufficient to infect a vervet monkey, even when metacyclic forms are not detectable in saliva [28].

PSSA-2 is rich in serine, threonine and tyrosine and has the potential to be highly modified, but no changes in electrophoretic mobility were observed when the protein was treated with λ phosphatase. In addition, no reactivity was detected with anti-phosphotyrosine, anti-sulphotyrosine or anti-OGlcNAc antibodies. One threonine residue (T_305_) in the cytoplasmic domain is phosphorylated, however. Mutation of T_305_ to alanine increased the electrophoretic mobility of the protein, indicating that it was aberrantly processed. In contrast, mutation of T_305_ to aspartic acid (which would mimic phosphorylation) had no effect on mobility. Taken together, this suggests that phosphorylation of T_305_ might be a prerequisite for further modifications. Phosphorylation of T_305_ was shown to depend on MAPK4, a kinase that was previously shown to protect procyclic forms against elevated temperatures [23]. Although T_305_ occurs in a context that makes it a possible substrate for a MAP kinase, it is not known if MAPK4 modifies it directly. Interestingly, PSSA-2 derived from cells deficient in MAPK4 migrated more slowly than PSSA-2 from wild type cells, suggesting alterations in post-translational modifications that differed from those described above. At present, the nature of all these modifications is unknown. There are two possible interpretations of these results. One possibility is that modifying enzymes in the cell can discriminate between threonine, phosphothreonine and alanine at this position. Additionally, MAPK4 may be required for the activity of auxiliary factors that are involved in post-translational modifications.

Deletion of the PSSA-2 gene had no discernible effect on the growth or morphology of procyclic forms in culture. Because of the dependence on MAPK4 for phosphorylation of T_305_, we also tested if the null mutant was temperature sensitive, but this was not the case (data not shown). Derivatives of the null mutant complemented with wild type PSSA-2 or PSSA-2(T_305_D) expressed the protein on the surface. This contrasted with PSSA-2(T_305_A), which was largely internal in the null mutant background, although it was expressed on the surface of wild type cells. These differences can be explained best by a model in which PSSA-2 forms homodimers or multimers. We postulate that in normal procyclic forms the association of a mutant polypeptide with a wild-type copy of PSSA-2 would be sufficient to transport it to the surface. In the absence of wild type PSSA-2, these mutant forms might not be able to interact efficiently with factors involved in cargo export from the endoplasmic reticulum or retention at the surface. The T_305_D mutant is not subject to these constraints, suggesting that aspartic acid functionally substitutes for phosphothreonine. There are several cysteine residues in PSSA-2, including one close to the C-terminal. If this particular cysteine residue is required for dimer formation, it could also explain why the truncated version is not exported to the surface since it would be unable to form disulphide bridges with full-length molecules. Although the T_305_D mutant localised correctly in both wild type and null mutant backgrounds, its expression slowed growth in both contexts. This effect was more pronounced in the null mutant background. Since cells grow normally without PSSA-2, one possibility is that the mutant protein, by mimicking permanently phosphorylated T_305_, sequesters factors away from wild type PSSA-2 and/or other molecules that are required for cell cycle progression.

In conclusion, PSSA-2 is the latest addition to a growing catalogue of surface molecules expressed by trypanosomes in their insect vector. Although it is a minor component of the coat compared to GPI-anchored proteins such as the procyclins, it has a clear role in the maturation of infection. Because of its structure, with a single membrane-spanning domain, PSSA-2 could potentially sense and transmit signals from the fly to the parasite and vice versa. It is also possible that the phosphorylation status of the protein, and its dependence on at least one MAP kinase, might contribute to the parasite's decision to proliferate, differentiate or migrate.

## Materials and Methods

### Trypanosomes

Procyclic forms of *T. brucei* AnTat 1.1 [29], [30] and genetically manipulated derivatives of it were cultured in semi-defined medium (SDM-79) [31], [32] supplemented with 10% heat-inactivated fetal bovine serum (FBS) at 27°C. Removing glycerol from the medium allows differentiation from early to late procyclic forms [1]. The MAPK4 and MAPK5 null mutants have been described previously [22], [23]. Bloodstream forms of AnTat 1.1 were isolated from mouse blood as described [15].

Stable transformation of procyclic form trypanosomes was performed as described previously [1] except that cytomix (120 mM KCl, 0.15 mM CaCl_2_, 10 mM K_2_HPO_4_ pH 7.6, 25 mM HEPES pH 7.6, 2 mM EDTA, 5 mM MgCl_2_) was used as the electroporation buffer. The following concentrations of antibiotics were used for selection of stable transformants: G418 25 µg ml^−1^, puromycin 1 µg ml^−1^ and phleomycin 2.5 µg ml^−1^.

To monitor generation times, cultures were propagated in SDM-79 supplemented with 10% FBS and 20 mM glycerol. The experiment was initiated with 2×10^6^ cells ml^−1^. Cells were counted every 24 hours and were kept in logarithmic growth by diluting them every day to 2×10^6^ ml^−1^.

### In vitro differentiation

Bloodstream form trypanosomes were resuspended at a density of 1–2×10^6^ cells ml^−1^ in SDM-79 supplemented with 10% FBS and 20 mM glycerol. Cells were triggered to differentiate by adding 6 mM cis-aconitate to the culture medium and incubation at 27°C [33].

### Isolation of total RNA from trypanosomes and Northern blot analysis

Total RNA extraction using the hot phenol method and Northern blot analysis were performed by standard procedures [34]. Ten micrograms of total RNA were loaded per lane. Blots were hybridised with probes corresponding to the coding regions of PSSA-2 or EP1. Radioactively labeled probes were generated using a Megaprime DNA labelling system (Amersham Biosciences, Buckinghamshire, UK) according to manufacturer's instructions. Blots were hybridised and washed under stringent conditions.

### Immunofluorescence analysis (IFA)

Cells were fixed for 15 minutes at room temperature in PBS containing 4% formaldehyde and 0.1% glutaraldehyde. After fixation, cells were permeabilised by adding 0.2% (v/v) Triton X-100. Unspecific antibody binding sites were blocked with PBS containing 2% (w/v) BSA. Fly-derived trypanosomes were fixed as described, with the exception that the cells were first air dried onto microscope slides and the permeabilisation step was omitted.

The HA epitope was detected with sc-805 (Santa Cruz Biotechnology, Santa Cruz, USA) diluted 1∶250 or 3F10 (Roche Applied Science, Basel, Switzerland) diluted 1∶500. For detection of EP and GPEET procyclins, the antibodies TRBP1/247 anti-EP [35], [36] and 5H3 anti-GPEET [37], gifts from Terry Pearson, University of Victoria, were diluted 1∶500. Anti-BiP antibody, kindly provided by Jay Bangs, University of Wisconsin, was diluted 1∶400.

The secondary antibodies anti-rabbit Alexafluor 568, anti-rabbit Alexafluor 488 and anti-mouse Alexafluor 488 (Molecular Probes, Eugene, OR) were diluted 1∶1000. Samples were analysed with a Nikon Eclipse E600 microscope connected to a Nikon DXM1200 CCD camera or with a Leica DM IRE2 inverted microscope connected to a Leica True Confocal Scanner.

### Cyclical transmission through tsetse flies

Pupae of *Glossina morsitans morsitans* were obtained from the Department of Entomology, Slovak Academy of Science, Bratislava. Transmission experiments were performed at the Swiss Tropical Institute, Basel, as described previously [38]. Flies were dissected 28–35 days after the infective blood meal, and midguts and salivary glands were analysed for the presence of trypanosomes. Infections were graded according to Ruepp et al. [38]. To complete the life cycle, salivary glands of flies with mature infections were injected intraperitoneally into mice.

### Constructs to generate GFP- and HA-tagged forms of PSSA-2

In order to amplify the full length and the truncated versions of PSSA-2, the A/C and A/B primer pairs were used respectively. The PCR was performed using genomic DNA from *T. brucei* AnTat1.1 as the template.

PSSA-2H (A): ACAAGCTTATGGCATCGAACAGCTC

PSSA-2SSII (B): TAGTCGACCATTGGATGAAGAGCC

PSSA-2SFII (C): TTGTCGACCTGTAGGATTTGGTTG

The restrictions sites used in cloning are underlined.

The coding sequences were cloned upstream of the coding sequence of a green fluorescent protein in pG-EGFPβ-ΔLII [39] via the *Hind*III and *Sal*I restriction sites, giving the plasmids pG-PSSA-2-EGFPβ-ΔII and pG-Δ^292–436^PSSA-2-EGFPβ-ΔII. In order to replace the 3′ UTR from pG-PSSA-2-EGFPβ-ΔII and pG-Δ^292–436^PSSA-2-EGFPβ-ΔII, the 3′ UTR/intergenic region of PSSA-2 was amplified by PCR using genomic DNA from *T. brucei* AnTat 1.1 with the following primers PSSA-2interB and PSSA-2interX (see following section).

The vectors and the PCR product were digested with *BamH*I and *Xba*I, and the 3′UTR sequences replaced, giving rise to pG-PSSA-2-EGFPβ-3′UTRPSSA-2 and pG-Δ^292–436^PSSA-2-EGFPβ-3′UTRPSSA-2.

To replace the GFP coding sequence with a haemagglutinin (HA) tag, the GFP sequence was released from pG-PSSA-2-EGFPβ-3′UTRPSSA-2 and pG-Δ^292–436^PSSA-2-EGFPβ-3′UTRPSSA-2 with *Sal*I and *BamH*I. The plasmids were repaired with Klenow to create blunt ends. Complementary primers for HA epitope tagging (HA-tag5-3/HA-tag3-5) were annealed then cloned into the prepared vectors.

HA-tag5-3: TACCCTTATGACGTACCAGACTATGCAGAAGGACGTGAACCT

HA-tag3-5: AGGTTCACGTCCTTCTGCATAGTCTGGTACGTCATAAGGGTA

The resulting clones were screened for inserts with the correct orientation and verified by sequencing. Δ^292–436^PSSA-2-HA contains a single HA tag while the full length PSSA-2-HA contains two copies of the tag.

### PSSA-2 null mutants and addbacks

To generate null mutants by homologous recombination, two targeting constructs were created: pPSSA-2-KO-neo and pPSSA-2-KO-phleo. In both vectors, the antibiotic resistance genes were cloned between the 5′ and 3′ flanking regions of PSSA-2. The flanking sequences were amplified by PCR from AnTat1.1 genomic DNA using the following oligonucleotides:

PSSA-2interH: AGAAGCTTTTCCTTGCGACGGCGTTA

PSSA-2interK: ACGGTACCGGGAGATTGGATACGATG

PSSA-2interB: ATGGATCCTTAACGCGGTTAGGATAG

PSSA-2interX: CCTCTAGAGTCCTTTACCTTGTCAAC

The restrictions sites used in cloning are underlined.

The 5′ region was amplified with the primer pair PSSA-2interK/PSSA-2interH and the 3′ region was amplified with the primer pair PSSA-2interB/PSSA-2interX.

For stable transfections, plasmids were cleaved with *Kpn*I and *Xba*I. To obtain a null mutant, cells were submitted to two rounds of transfection.

A construct encoding an untagged version of PSSA-2 was generated from pG-PSSA-2-EGFPβ-3′UTRPSSA-2 in two steps. In a first step, the neomycin-resistance gene was replaced by puromycin-resistance [38]. In a second step, the open reading frame for PSSA-2-EGFP was replaced by a PCR product generated using the oligonucleotides PSSA-2H (see section below) and revPSSA2stop (TTGTCGACCTACACTGTAGGATTTGG).

### Site-directed mutagenesis

To mutate thr_305_ (T_305_) in PSSA-2-HA to alanine (pG-T_305_A/PSSA-2-HA-3′UTRPSSA-2) or aspartic acid (pG-T_305_D/PSSA-2-HA-3′UTRPSSA-2), we designed primers containing mutated codons. The triplet corresponding to T_305_ ACA was mutated to a GCA triplet coding for alanine or to a GAT triplet coding for aspartic acid.

Complementary primers were used in two separate PCRs with an outer flanking primer, in order to amplify the two halves of PSSA-2. E/B* and A*/F primer pairs were used to mutate threonine to alanine. The threonine to aspartic acid mutation was generated with E/D and C*/F primer pairs. The two PCR products were combined as a template for the second PCR, which was done using the outer flanking primers (E/F).

Complementary overlapping mutagenic primers:

PSSA-2.thr-ala.fw (A*): gatcctgat**gca**cctcagggggatgg

PSSA-2.thr-ala.rv (B*): CCATCCCCCTGAGG**TGC**ATCAGGATC

PSSA-2.thr-asp.fw (C*): GATCCTGAT**GAT**CCTCAGGGGGATGG

PSSA-2.thr-asp.rv (D): CCATCCCCCTGAGG**ATC**ATCAGGATC

The mutated codons are shown in boldface type.

Outer flanking primers:

PSSA-2H (E): ACAAGCTTATGGCATCGAACAGCTC

PSSA-2interX (F): CCTCTAGAGTCCTTTACCTTGTCAAC

The restrictions sites used in cloning are underlined.

The mutated sequences created by PCR were used to replace the wild type sequence in the plasmid pG-PSSA-2-HA-3′UTRPSSA-2. All constructs were sequenced before being used for transfection. For stable transformations, plasmids were cleaved with *Spe*I in order to integrate the plasmid upstream of a procyclin locus. Transformants were selected with G418 as above. Introduction of an ectopic copy of PSSA-2 into the null mutant was achieved by using modified versions of the plasmids described above, in which the neomycin resistance gene was replaced by puromycin. For stable transfections, plasmids were cleaved with *Spe*I.

### Immunoprecipitation and immunoblot analysis

For each immunoprecipitation 1–2×10^8^ cells were harvested by centrifugation at 1300 g, 10 minutes at 4°C. Cells were washed once in cold PBS and the pellet resuspended in 1 ml of Lysis Buffer (20 mM Tris-HCl pH 7.5, 2 mM EDTA, 2 mM EGTA, 1% (v/v) Triton X-100) supplemented with protease inhibitor cocktail (Roche Applied Science) according to manufacturer's instructions. The suspension was passed through a 27G needle three times, centrifuged at 16000 g for 10 minutes at 4°C and the supernatants were transferred to fresh tubes. 20–25 µl of haemagglutinin-tagged agarose beads (anti-HA affinity matrix, Roche Applied Science) were washed twice in PBS and incubated in Lysis Buffer, 2% (w/v) casein for 1 hour at room temperature on an end-over-end rotating platform. The matrix was then washed three times with Lysis Buffer and added to the supernatants. Matrix and supernatants were incubated at room temperature on a rotor for 2 hours, after which the matrix was pelleted and washed four times in Lysis Buffer. The washed matrix was then heated at 50°C for 5 minutes in 30 µl Laemmli Buffer [40]. 10 µl aliquots were used for immunoblot analysis. Following SDS-PAGE, samples were transferred to Immobilon-P (Millipore, Bredford, MA, USA). Membranes were blocked with TBS containing 0.05% (v/v) Tween20 2% (w/v) BSA followed by overnight incubation at 4°C with the primary antibodies. The following antibodies were used: anti-HA antibody (12CA5, Roche Applied Science) diluted 1∶1000; anti-phospho-threonine-proline antibody (Cell Signaling, Beverly, MA, USA) diluted 1∶500; anti-phospho-tyrosine antibody (Cell Signaling) diluted 1∶1000; TRBP1/247 anti-EP antibody [35], [36] diluted 1∶2500; 5H3 anti-GPEET antibody [37] diluted 1∶1000. HRP-conjugated secondary antibodies anti-mouse or anti-rabbit (DAKO, Glostrup, Denmark) were usually diluted 1∶3000, with the exceptions of anti-EP and anti-GPEET where the secondary antibodies were diluted 1∶10000. Membranes were exposed to Enhanced Chemiluminescent Substrate (Pierce Rockford, USA) and chemiluminescent signals captured on X-ray film.

### λ PPase and PNGase F treatment

Both reactions were performed with kits from New England BioLabs (Beverly, MA, USA). For λ PPase treatment 1–2×10^7^ cells were pelleted and washed once in PBS, resuspended in 40 µl of H_2_O, 5 µl of 10× λPPase Reaction Buffer and 5 µl of 10× MnCl_2_ stock solution. After pre-incubation at 30°C for 30 minutes, 200 U of λPPase were added and the reaction incubated for another 30 minutes. For PNGase F treatment, 1–2×10^7^ cells were pelleted and washed once in PBS. Cells were resuspended in 36 µl of H_2_O and 4 µl of 10× Glycoprotein Denaturing Buffer and boiled for 10 minutes. After cooling to room temperature, 5 µl of 10% NP40, 5 µl of 10× G7 Reaction Buffer and 500 U of PNGase were added and the lysates were incubated at 37°C for 2 hours. As negative controls, cells were treated in the same way but λPPase or PNGase were omitted. An equal volume of H_2_O was used as a replacement for the enzyme.

## Supporting Information

Figure S1 Analysis of PSSA-2 addbacks
(1.36 MB PDF)

Figure S2 Localisation of PSSA-2 T305A in a null mutant background
(0.56 MB TIF)
