# Supplementary material for: PSSA-2, a Membrane-Spanning Phosphoprotein of Trypanosoma brucei, Is Required for Efficient Maturation of Infection
Source: PLoS One. 2009 Sep 17;4(9):e7074. doi: 10.1371/journal.pone.0007074 (PMC2739429; doi:10.1371/journal.pone.0007074)
Supplement: Figure S1 — Analysis of PSSA-2 addbacks (1.36 MB PDF) [file pone.0007074.s001.pdf]

Supplemental figure S1a

Integration sites of PSSA-2 addbacks

The construct is designed to integrate upstream of a procyclin locus. Genomic DNA from each clone was analysed in two PCR reactions with a primer in the plasmid backbone (T3) and primers specific for EP1 on chromosome 10 (E-10) and and GPEET on chromosome 6 (G-6), respectively.

Addback clones 1 and 2 both have the PSSA-2 gene integrated on chromosome 10.
Plasmids that have not integrated give no PCR product in either reaction (-). Cell lines with plasmids integrated on chromosome 6 and 10 (theoretically possible, but unlikely) would give a product in both reactions.

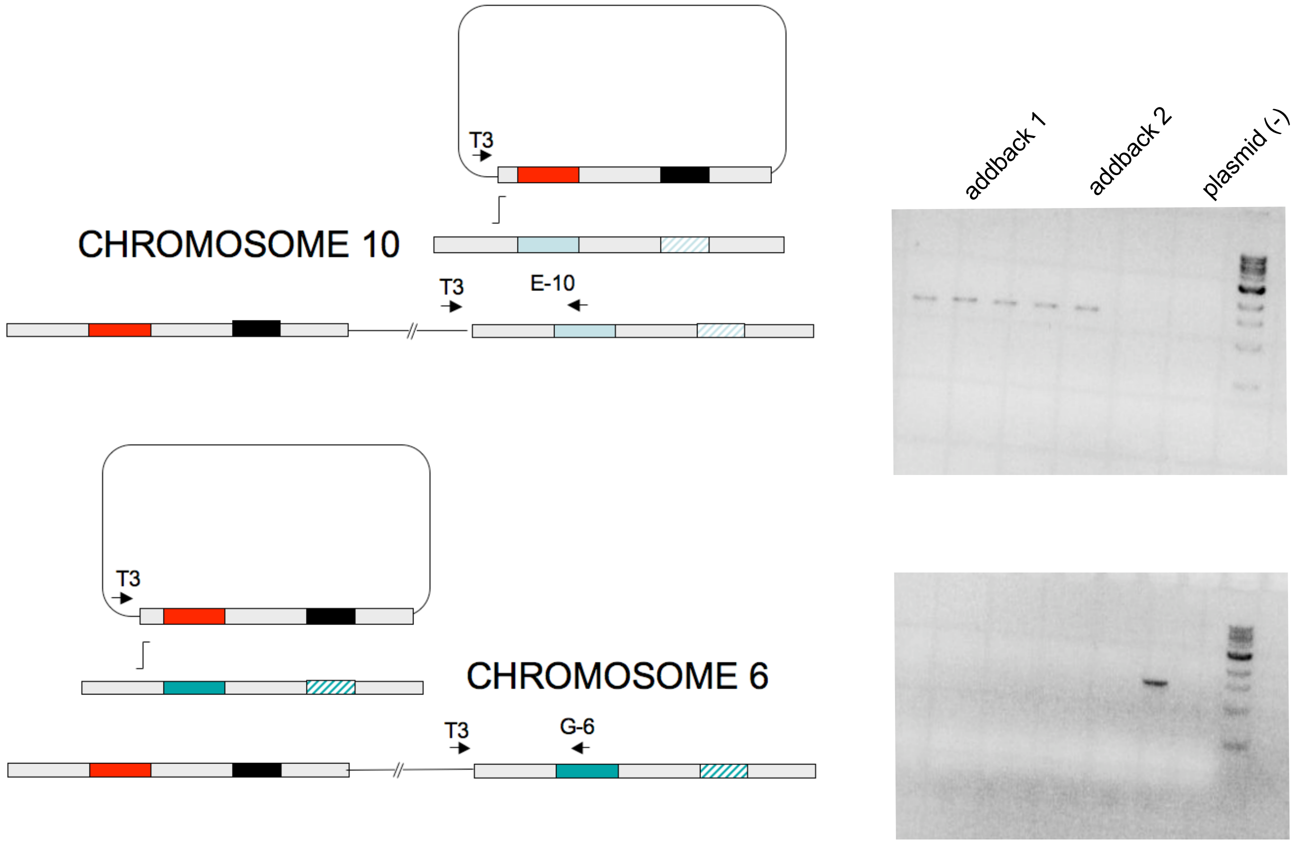

Primers
T3:   AATTAACCCTCACTAAAGGG
E-10: TAAGGCATTAGAAAGGATGTCAC
G-6: AACGGATAAAATAAAGGATAGAAC

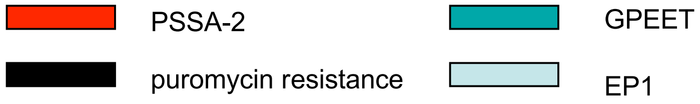

Supplemental figure S1b

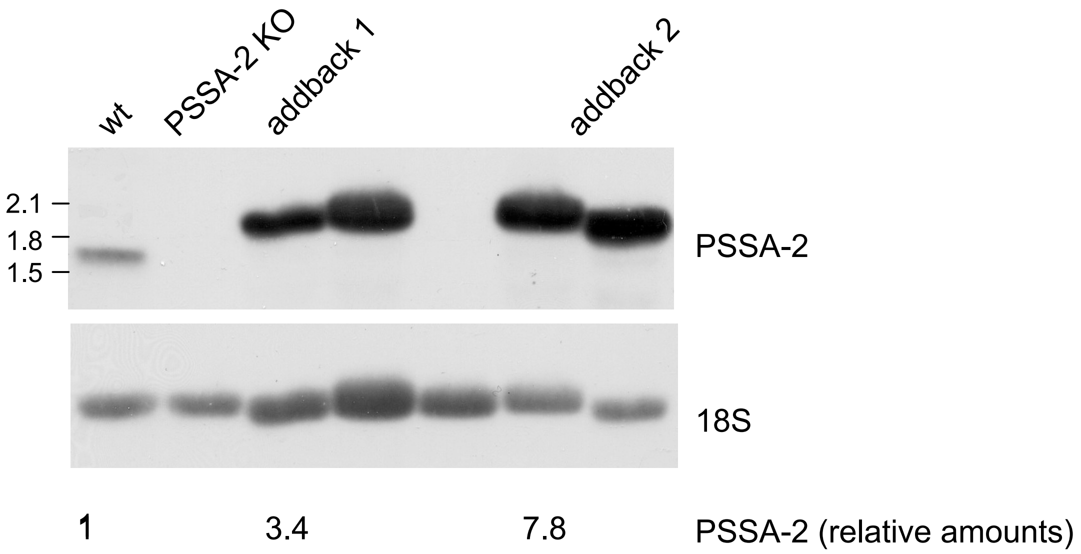

Northern blot analysis to determine relative expression levels of PSSA-2 mRNA.

Total RNA was isolated from wild type AnTat 1.1, the PSSA-2 null mutant and five independent “addback” clones derived by transfection of the null mutant with a construct encoding an untagged version of PSSA-2 (see Materials and Methods). Ten micrograms total RNA were loaded per lane and hybridised sequentially with probes corresponding to the coding region of PSSA-2 and an oligonucleotide complementary to18S rRNA. The positions and sizes of rRNAs (in kb) are marked on the left. The ectopically expressed copies of PSSA-2 give rise to a longer transcript than the wild type.
Quantitation was performed using a PhosphoImager (Molecular Dynamics). Relative amounts of PSSA-transcripts (normalised against 18S) are shown for the 2 clones used in this study.
