# Supplementary figures and images for: PSSA-2, a Membrane-Spanning Phosphoprotein of Trypanosoma brucei, Is Required for Efficient Maturation of Infection
Source: PLoS One. 2009 Sep 17;4(9):e7074. doi: 10.1371/journal.pone.0007074 (PMC2739429; doi:10.1371/journal.pone.0007074)

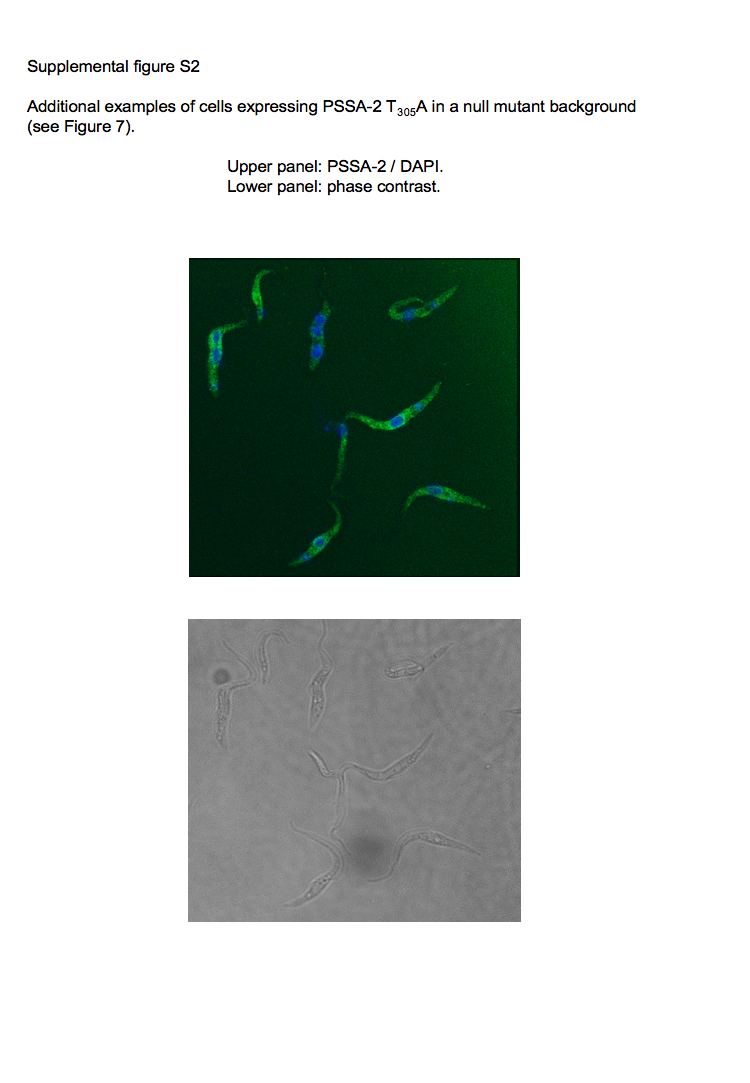

Supplement: Figure S2 — Localisation of PSSA-2 T305A in a null mutant background (0.56 MB TIF) [file pone.0007074.s002.tif]
